# The Valorization of Banana By-Products: Nutritional Composition, Bioactivities, Applications, and Future Development

**DOI:** 10.3390/foods11203170

**Published:** 2022-10-11

**Authors:** Fanglei Zou, Chunming Tan, Bo Zhang, Wei Wu, Nan Shang

**Affiliations:** 1 College of Engineering, China Agricultural University, Beijing 100083, China; 2 Beijing Laboratory for Food Quality and Safety, College of Food Science and Nutritional Engineering, China Agricultural University, Beijing 100083, China; 3 Key Laboratory of Precision Nutrition and Food Quality, Department of Nutrition and Health, China Agricultural University, Beijing 100083, China

**Keywords:** banana by-products, composition, active ingredients, agricultural waste utilization, development

## Abstract

Bananas are among the world’s main economic crops and one of the world’s most-selling fresh fruits. However, a great deal of waste and by-products is produced during banana harvesting and consumption, including stems, leaves, inflorescences, and peels. Some of them have the potential to be used to develop new foods. Furthermore, studies have found that banana by-products contain many bioactive substances that have antibacterial, anti-inflammatory, and antioxidant properties and other functions. At present, research on banana by-products has mainly focused on various utilizations of banana stems and leaves, as well as the extraction of active ingredients from banana peels and inflorescences to develop high-value functional products. Based on the current research on the utilization of banana by-products, this paper summarized the composition information, functions, and comprehensive utilization of banana by-products. Moreover, the problems and future development in the utilization of by-products are reviewed. This review is of great value in expanding the potential applications of banana stems, leaves, inflorescences, and peels, which will not only help to reduce waste of agricultural by-product resources and ecological pollution but will also be useful for the development of essential products as alternative sources of healthy food in the future.

## 1. Introduction

**Figure 1:**
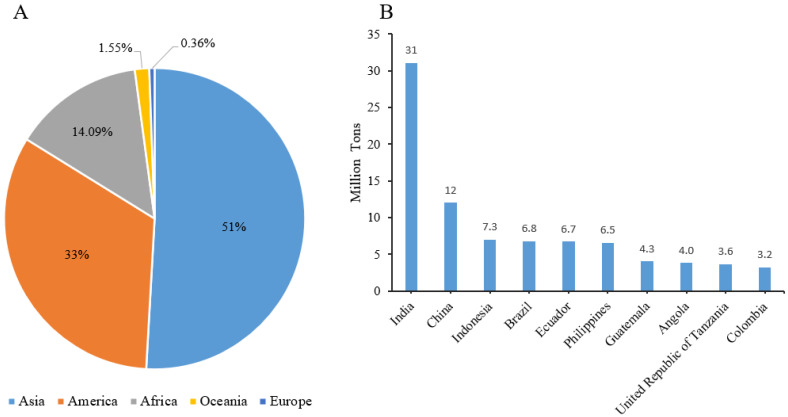
(**A**) Percentage data of banana production by region and (**B**) The top 10 largest banana producing countries. Adapted from [5].

**Figure 2:**
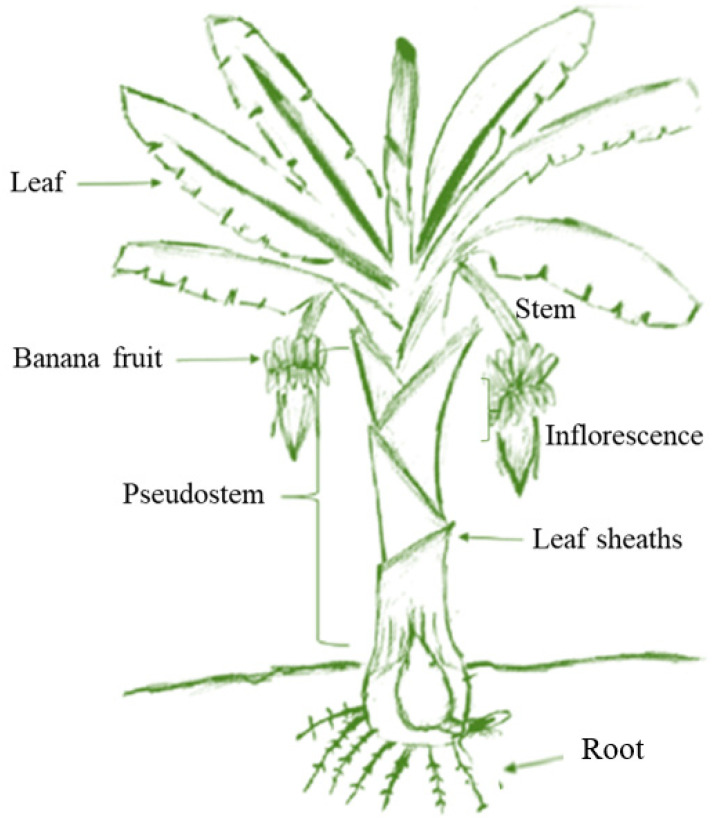
The structure of banana plant. Adapted from [11].

Bananas are classified as part of ‘Magnoliophyta’, class ‘Liliopsida’, order ‘Zingiberales’, family ‘Musaceae’, and genus ‘Musa’ [1] and are the fourth largest food crop in the world. In the past two decades, banana production has continued to expand, rising from over 70 million tons in 1999 to around 121 million tons in 2021. Bananas are mainly produced in Asia, America, and Africa (Figure 1A), and the world’s top banana growers are India, China, Indonesia, Brazil, Ecuador, and the Philippines (Figure 1B). Bananas are grown mainly for their fruit, but banana trees also contain stems, leaves, pseudostems, leaf sheaths, inflorescences, and other parts, as shown in Figure 2. Therefore, banana farms generate large amounts of underutilized by-products and waste. It has been reported that each ton of bananas collected generates about 4 tons of waste, including 100 kg of waste fruit, 3 tons of pseudostems, 160 kg of stems, 480 kg of leaves, and about 440 kg of inflorescences and skins [2]. Among them, banana peel, one of the by-products of bananas, accounts for about 35% of the total weight of bananas, about 40 million tons of peel are produced per year. Most of these peels are often disposed of in landfills or next to other waste [3]. Ineffective agricultural waste management practices leave a large amount of this valuable raw material unused and even cause significant environmental damage [4]. Transforming banana by-products into useful commodities would enhance agricultural development.

Recently, there has been a change in how banana by-products are managed, with a focus on transforming them into value-added goods. The diverse bioactive chemicals with potential health-promoting benefits have reignited interest in banana by-products. The use of banana by-products for value-added purposes to meet demand in areas such as food alternatives, feed, renewable energy, textiles, and fiber composites is a constant challenge [6].

Banana stems and leaves can be used as resources for, e.g., fertilization, fodder, energy, and fiber, including food applications such as coloring, flavoring, livestock feed, and non-food applications such as wastewater treatment, biofertilizers, textiles, paper, and composites [7,8]. Banana inflorescence has been reported to have a variety of nutritional components, being rich in phenolics and other functional substances such as proteins, dietary fibers, and enzymes that have been demonstrated to have potential health benefits [9]. Banana peel also has a considerable amount of dietary fiber and phenolic chemicals, as well as antioxidant, antibacterial, and antimicrobial properties [10]. This encourages its use in applications such as nutritional products and pharmaceuticals. This paper provides an overview of the nutritional components, bioactivities, and utilization of banana by-products, which might enhance their potential application in the food industry and non-food industry, contributing to a significant reduction in agricultural waste. In addition, the information provided in this review can be used to develop or commercialize banana compositions into novel food products and value-added products.

## 2. Banana Stem and Leaves

### 2.1. Fraction of Banana Stems and Leaves

#### 2.1.1. Bio-Oil

Bio-oil is a high-density oxygenated liquid that can be used to replace liquid fossil fuels. Due to its comparatively high density (1200 kg/m^3^), bio-oil is thought to have financial advantages over other thermal conversion processes [12]. The slow pyrolysis of bio-oil produces more biochar, which can be used as a soil amendment to improve soil quality and sequester carbon, yielding higher environmental benefits. On the other hand, the rapid pyrolysis of bio-oil has the ability to produce higher-value products.

Taib et al. [13] conducted a study to determine whether banana pseudostem (BPS) could be rapidly pyrolyzed to produce high-quality bio-oil. The results indicated that the BPS bio-oil had better performance and production than other kinds of biomass bio-oil, and the groundwork for future applications was laid by pre-treating BPS with ash reduction to improve the liquid quality.

#### 2.1.2. Minerals

Banana stems are rich in macronutrients such as potassium (K), calcium (Ca), phosphorus (P), magnesium (Mg), and sodium (Na): Potassium and sodium together play an important role in maintaining the acid–base balance in the body, calcium and phosphorus are the most important components of human bones and teeth, and magnesium has a role in the body in maintaining muscle contractility and nerve stress [14]. Banana stems and leaves are also reported to be rich in carotene, nicotinic acid, riboflavin, thiamin, and other vitamins. It is an agricultural waste resource with an integrated nutrient profile [15]. Amini-Khoozani et al. [16] reported that the total dietary fiber, macronutrients (K, Ca, P, Mg, and Na), and mineral content of bread increased when freeze-dried whole green banana flour was used as an up to 30% substitute for wheat flour.

#### 2.1.3. Dietary Fiber

Bananas are rich in dietary fiber, and they have received attention in recent years due to their significant health-promoting effects [17]. Dietary fiber is composed of cellulose, hemicellulose, pectin, and lignin and is a non-starchy polysaccharide that cannot be digested or absorbed by the human gastrointestinal tract. Banana stems are a great source of fiber that aid in detoxification and the management of obesity [14]. Li et al. [18] reported that banana pseudostem flour has high dietary fiber and has potential as a functional food additive for high dietary fiber foods.

#### 2.1.4. Polyphenols and Flavonoids

A definite drawback of the many techniques recognized for gingival depigmentation is gingival repigmentation. A method that can lengthen the time until pigmentation reappears is required to get around this. Numerous studies using antioxidant-rich plant extracts have demonstrated their antimelanogenic effects. Sowmya et al. [19] treated melanocytes with ethanolic extract of banana stems and found a significant reduction in melanin content, suggesting that ethanolic extract of banana stems has a good inhibitory effect on melanin and is a potential periodontal decolorizing agent.

### 2.2. Comprehensive Utilization of Banana Stems and Leaves

#### 2.2.1. Fertilizer Utilization

**Figure 3:**
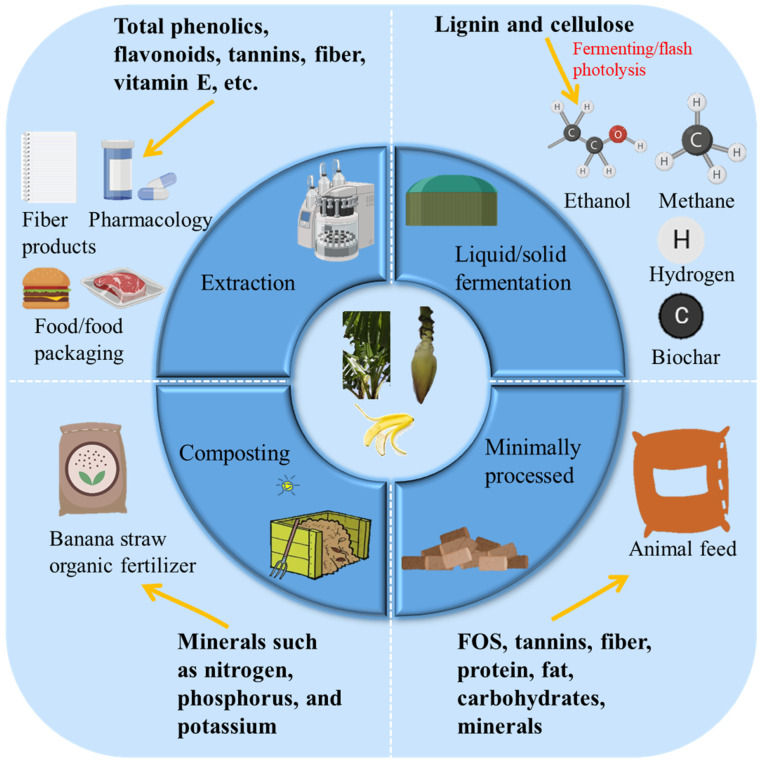
Integrated use of banana by-products.

The potential value of banana by-products is driving the development of processing technologies to create products for the food and non-food industries (Figure 3). Composting has been an important way for farmers to improve soil fertility for thousands of years. Banana stems and leaves contain a high level of water-soluble organic matter, and these organic components are easily degraded, they have a suitable carbon-to-nitrogen ratio and is a good material for aerobic composting [20]. The functions of banana stem and leaf components and the application of each component are shown in Table 1.

In the study by Ding et al. [24], straw organic fertilizer increased yields by more than 3.50% compared with regular organic fertilizers, with an economic benefit of CNY 1800/ha. De Siqueira et al. [25] reported that the substrates of banana stems and leaves or in combination with other wastes such as wheat bran, hay, soybean straw and cotton textiles were more beneficial to the growth of Amanita mushrooms.

#### 2.2.2. Feed Utilization

According to the study of Amarnath and Balakrishnan [26], banana stems and leaves are a good source of ruminant feed. Gregory et al. [27] chopped fresh banana leaves and dried them at 40 °C and fed them to sheep artificially infected with parasites, and they found that the tannins contained in the banana leaves inhibited the growth of serpentine woolly nematode eggs, providing a method of parasite exclusion. In addition, Marie-Magdeleine et al. [28] reported banana leaves as good feed because of their non-significant differences in crude protein, ATP and fiber content compared with good quality feed, which can be used to sustain animals during seasonal feed shortages. In addition, banana rhizome has been indicated for the treatment of rabbits with coccidiosis: oral crushed banana rhizomes significantly reduced fecal worm egg counts over two weeks of treatment [29].

Oleforuh-Okoleh et al. [15] fed broilers with a diet containing 50 g/kg banana leaf powder, and the results showed that all traits of broilers in the banana leaf treatment group were better than those in the control group. The reason for this result may be that banana leaves contain a large number of nutrients and high levels of fructo-oligosaccharides, which can promote the growth of beneficial bacteria in the broiler intestinal tract and secrete vitamins and digestive enzymes, thereby improving the overall absorption capacity of broilers.

#### 2.2.3. Biomass Energy Utilization

The stems and leaves of bananas are also potential substrates for biofuel production by co-culture of cellulolytic *Clostridium thermophilus* and *Clostridium thermosaccharolyticus* [20]. It is reported that the pseudostems of banana can be used to produce 17.1 g/L of ethanol. In general, banana pseudostems can be pretreated with a mixture of enzymes or chemicals that can lead to hydrolysis of the substrate. Treated banana pseudostems have also been shown to produce ethanol during fermentation [19]. Taer et al. [30] successfully synthesized banana stem fiber-activated carbon without added binder by using zinc chloride activator as a supercapacitor electrode. That study showed that banana stem fiber has the potential to be developed as a carbon electrode for supercapacitor batteries.

#### 2.2.4. Fiber Products

The chemical composition of banana fiber is mainly cellulose, hemicellulose and lignin; with its biodegradability and good chemical and mechanical properties, banana fiber has potential applications in the sanitary napkin, textile, pulp and paper industries [11], its chemical properties have many similarities with traditional fibers. Banana fiber is resistant to alkali, chloroform, acetone, formic acid, phenol and petroleum ether. Moreover, compared with cotton and chemical fibers, banana fiber is lustrous and has high water absorption, low irritation to the human body, and no negative impact on the environment [7,31,32].

For decades, banana fibers obtained from banana pseudostems have been used as a raw material for the production of textiles for traditional handicrafts and clothing. Currently, the global export value of the textile and clothing industry is increasing year after year, which means that there is a huge demand for textile fiber materials. The enzymatic degumming of natural plant fibers using microbial strains is a promising method for processing textile fibers [33]. Another commercial use of banana stems is papermaking, where natural wood has become more expensive due to limited supply [34]. Ogunsile et al. [35] used banana by-products as one of the raw materials for pulp production, with approximately 40% of the pulp from the pseudostems and leaves, and pulping parameters such as pulping time, temperature, and pH had great impacts on yield. Banana fiber paper is reported to have very low water absorption, which makes it stronger and more resistant to water than wood pulp paper [21].

## 3. Banana Inflorescence

### 3.1. Fraction of Banana Inflorescence

#### 3.1.1. Protein

The ratio of essential amino acids to nonessential amino acids required by the human body in banana inflorescences is 0.54 [36], which is greater than the amount advised for adults by the WHO. Bhaskar et al. [37] found that the protein content of banana inflorescences was 12.50%, which include high concentrations of aspartic acid, glutamic acid, alanine, arginine, leucine, proline, and serine. Sitthiya et al. [9] used ultrasonic-assisted alkaline extraction to extract proteins from banana inflorescences containing various polypeptides and amino acids; these proteins have significant antimicrobial potential against Gram-positive and negative bacteria.

#### 3.1.2. Carbohydrates

Research on the carbohydrates in banana inflorescences is still in its infancy. However, a large amount of carbohydrates were detected [9]. Bhaskar et al. [37] studied polysaccharides isolated from banana inflorescences, and the results showed the presence of a polysaccharide of the arabinogalactan type in the water-soluble ingredients, a polysaccharide of the rhamnogalacturonan type in the pectic fraction, and a polysaccharide of the xyloglucan and arabinoxylan type in the hemicellulose ingredients. These components have different antioxidant capabilities. However, whether banana inflorescences contain other polysaccharides and whether their functional features are similar to the reported compositions for other part of banana deserves further study.

#### 3.1.3. Lipids

According to Oliveira et al. [38], banana inflorescence also contains lipophilic chemical components such as long-chain aliphatic alcohols, triterpenes, and fatty acids. Additionally, about 12% of the lipophilic elements in the inflorescence were made up of sterols, of which campesterol, β-sitosterol and sitosterol were the main ones. However, other sterols include 24-methylenecycloartanol, cycloartenol, cholesterol, cycloeucalenol, and 31-norcyclolaudenone.

Although there were reports of different fatty acid levels in banana inflorescences, their primary fatty acids were somewhat similar. Sheng et al. [39] found that unsaturated fatty acids (UFAs) such as linoleic acid, oleic acid, and alpha-linolenic acid accounted for more than 60% of the total fatty acids in banana inflorescences. Linoleic acid has the effect of lowering cholesterol in the body and preventing atherosclerosis [36]. This shows that banana inflorescences can be an important source of healthful UFAs, which may lower the chance of cardiovascular disease [40].

#### 3.1.4. Minerals

The mineral makeup of banana inflorescences is balanced between macro and micro elements. Among them, potassium is the most abundant macronutrient, ranging between 33.52 and 55.2 mg/kg [41]. The next most abundant macronutrients are magnesium and calcium [33,36,42], but the inflorescences also have significant concentrations of iron, copper and zinc in terms of its micro elements, while iron is an essential element in human physiology and is one of the main raw materials for hemoglobin [19]. Therefore, banana inflorescences are an important source of minerals for human beings [41].

#### 3.1.5. Polyphenols

It has been discovered that the banana inflorescence is abundant in polyphenols: phenolic acids such gallic acid, protocatechuic acid, p-hydroxylbenzoic acid, syringic acid, vanillic acid, gentisic acid, caffeic acid, vanillin, and ferulic acid were found [36,43,44,45]. In addition, some of the flavonoids discovered in the inflorescence include catechin, epicatechin, quercetin, and rutin [36,43,44,45]. Indeed, phenolic and flavonoid concentrations and extract antioxidant capacities have been discovered to be closely correlated, suggesting that these groups of secondary metabolites may be in charge of the bioactivity.

### 3.2. Biofunction

**Figure 4:**
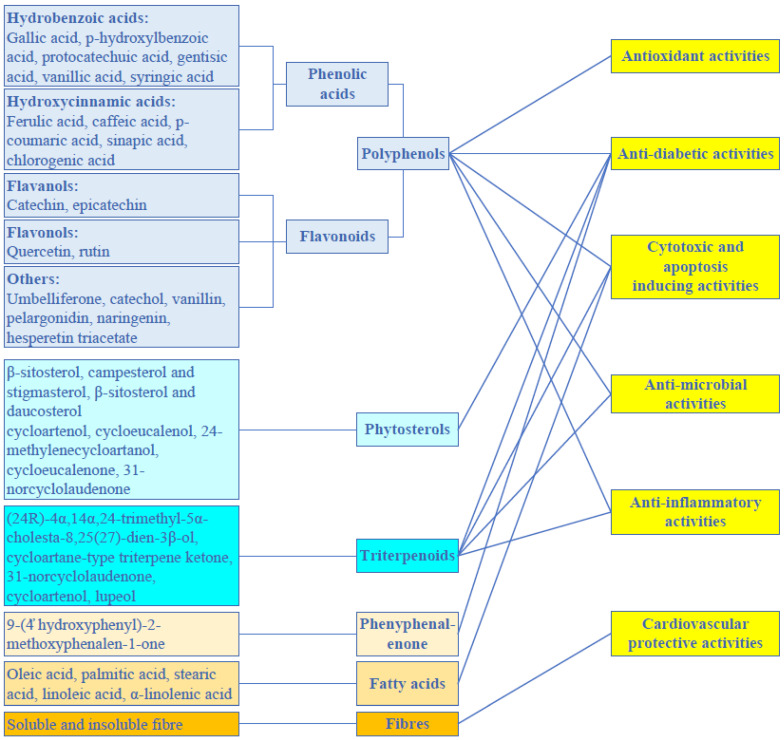
Relationship between the banana inflorescence’s phytochemical components and its practical characteristics. Adapted from [40].

Banana inflorescences have been shown to exhibit a variety of biological functions with health-promoting potential. Particularly, the antioxidant properties of the inflorescence have drawn much interest. In addition, it has been proved that the inflorescence may possess antidiabetic, anticancer, cardioprotective, and other biologically active functions, which will be covered in the sections that follow. Figure 4 shows a scientifically supported relationship between the biofunctional ingredients of the inflorescence and its functional activities.

#### 3.2.1. Antioxidant Activities

Many medical studies show that consuming antioxidants reduces the risk of diseases like diabetes, cancer, and cardiovascular disease [46]. Banana inflorescence and its extract can be utilized as antioxidant sources of food products. Banana inflorescence extracts can inhibit the oxidation process, prevent molecular destabilization of DNA, and control various heart problems [36,47,48].

Schmidt et al. [49] investigated the effects of extraction methods (conventional and ultrasound-assisted) on the antioxidant capacity of banana inflorescences, and according to their findings, the best conditions for the extraction of antioxidants were those that involved stirring for 30 min at a temperature of 60 °C while using aqueous ethanol (*v*/*v*) as the solvent. The application of green technologies for extraction has been studied by some researchers. For instance, Madeline Correa et al. [50] optimized the conditions for extracting antioxidants using the supercritical fluid extraction method. They discovered that extracts made at greater pressures (25.0 MPa) and temperatures (353.15 K) exhibited the greatest activity. The antioxidant capacity of inflorescence extracts can be evaluated using a multi-mechanism antioxidant assay. These antioxidant assays were used to evaluate the capacity of chemical constituents in inflorescence extracts to scavenge free radicals, such as 2,2′-azinobis-(3-ethylbenzthiazoline-6-sulphonate (ABTS), 2,2-diphenyl-1-picrylhydrazyl (DPPH), nitric oxide and superoxide anion. Table 2 lists the biofunctions of banana inflorescences and their applications. The antioxidant capacity of the low molecular weight components of inflorescences was studied in depth by Lau et al. [40]. These metabolite-rich extracts/fractions have been shown to reduce oxidative stress by scavenging free radicals.

#### 3.2.2. Antidiabetic Activities

In drug discovery, the ability of extracts or compounds to block carbohydrate-hydrolyzing enzymes is typically seen as a sign of their potential antidiabetic effect. The isolated components of the banana inflorescence have been shown to have variable degrees of inhibitory action on α-amylase and α-glucosidase. For example, Ramu et al. [53] found that the ethanol extract of banana inflorescence was more effective than acarbose at inhibiting α-glucosidase but less effective at inhibiting α-amylase. The primary chemicals in the extract, umbelliferone and lupeol, were shown to inhibit both enzymes in a noncompetitive way. Bhaskar et al. [60] studied the antidiabetic efficacy of banana inflorescences thoroughly, and the results indicated that the rats fed the banana inflorescences had decreased urine sugar and blood sugar levels; the activity of the lactase and sucrase were also improved by feeding. Bhaskar et al. found that banana inflorescence reduced glomerular filtration rates in diabetic rats and prevented fructosamine formation and advanced glycation end products (AGEs) in rat liver, serum, and kidney. The extensive array of secondary metabolites blocking important enzymes, and acting on various molecular targets implicated in diabetes is credited to the banana inflorescence’s possible anti-diabetic action.

#### 3.2.3. Anti-Cancer Activities

Studies have shown that banana inflorescence extracts have potential anticancer effects on a group of human cancer cells. Two human colorectal cancer cell lines, HT29 and HCT116, were especially sensitive to the ethanol extracts of the banana inflorescence when compared with other cancer cell [48,56,61]. The extracts were less cytotoxic to human umbilical vein endothelial cells, indicating that the extracts’ cytotoxic active compounds being highly selective.

However, the exact molecular mechanism of action of the cytotoxic constituents in the inflorescence is unknown. Preliminary findings suggested that cell death may be caused by the induction of apoptosis [61]. In the HT29 cell experiment, an ethanol extract of banana inflorescences caused morphological alterations consistent with apoptotic cell death, including the loosening of cell-to-cell contact, membrane blebbing, and cell shrinkage [48].

#### 3.2.4. Cardiovascular Protective Activities

Research showed that the expression of pro-inflammatory genes is increased by oxidized low density lipoprotein (LDL), which leads to monocyte recruitment into the vascular endothelial cells of a defective blood vessel wall that has been damaged by free radicals. Inhibiting LDL oxidation is critical in the treatment of cardiovascular disease and atherosclerosis. Arun et al. [43] found that the extracts of banana inflorescence in methanol and ethyl acetate significantly reduced LDL oxidation in a dose-dependent manner, with IC_50_ values of 169.52 and 217.45 μg/mL, respectively. The angiotensin I-converting enzyme (ACE) converts angiotensin I to angiotensin II, a strong vasoconstrictor that is important for blood pressure management. Angiotensin II has been linked to the advancement of vascular problems in diabetes as a key activator of insulin resistance. Therefore, ACE inhibition appears to be a new treatment option for diabetes management.

One study showed that the soluble dietary fiber from inflorescences had a higher cholesterol-absorbing capacity than the insoluble dietary fiber [43]. The present results indicated that banana inflorescence may exercise its cardiovascular protective action by blocking several targets, although this is still a work in progress. Future research should focus on finding inhibitors of these targets.

#### 3.2.5. Other Biological Activities

Apart from the biological activities already mentioned, the banana inflorescence has been proven to have other biofunction, such as the treatment of prostate enlargement and the improvement of urinary disorders [62]. Banana inflorescence extracts have shown varying degrees of antibacterial activity. They are active against Gram-positive (e.g., *Bacillus cereus, Bacillus subtilis*, and *Staphylococcus aureus*) and Gram-negative bacteria (e.g., *Escherichia coli*, *Salmonella typhimurium*, and *Pseudomonas aeruginosa*) and some pathogenic fungi (e.g., *Cryptococcus albidus* and *Candida albicans*) [57,58,63]. Tin et al. [63] showed that the effectiveness of extracting anti-microbial chemicals from banana inflorescences was affected by extraction parameters such as extraction temperature, extraction time, and sample-to-solvent ratio. Additionally, compared with extracts produced using newly developed ultrasonic and microwave-assisted extraction techniques, extracts produced using traditional extraction techniques, such as a shaking water bath and reflux, had stronger activity. According to their findings, methanol as the extraction solvent, sample drying at 50 °C, a sample-to-solvent ratio of 1:10 (*w*/*v*), and a period of 3 h offered the ideal conditions for extracting chemicals with anti-microbial activity from the banana inflorescence. Red blood cells were used for testing. Divya et al. [59] discovered that the water extract of the inflorescence had a membrane-stabilizing effect; these extracts might also help to stabilize lysosomal membranes and could help to inhibit the inflammatory response. In addition, banana inflorescences have an immunomodulatory effect on the complement system [64]. Divya et al. [59] also investigated the anti-obesity potential of the inflorescences and discovered that inflorescence extracts inhibited pancreatic lipase, implying that they may have lipid-lowering properties.

Although banana inflorescence extracts have been shown to have many health-promoting biological activities; however, there is still a lack of information from clinical studies to support its alleged health benefits. Additional mechanistic research on some of the extracts, components, and metabolites existed in banana inflorescences could shed more light on their potential health-promoting qualities.

### 3.3. Comprehensive Utilization of the Banana Inflorescence

#### 3.3.1. Pharmacological Applications

Like other parts of the banana plant, the inflorescence has been used to cure a number of diseases. Banana inflorescence has been used for a variety of medical purposes around the globe, generally for the treatment of ailments like diabetes, bronchitis, ulcers, heavy menstruation, and menstrual cramps in the past. For instance, indigenous healers in India think that the juice from the banana inflorescence is helpful for reducing blood sugar levels, and banana inflorescence can be made into a preparation used to treat diabetes [65]. Indigenous people from Brazil use inflorescences to prepare syrups that are then used as expectorants to treat respiratory diseases [64].

#### 3.3.2. Food Applications

Banana inflorescence extract can be employed as a nutritive supplement in food products. Most often, banana inflorescence or banana inflorescence extract used as a food additive. However, it has a significant impact on the physical, chemical, and sensory properties of food products, the appropriate amount of inflorescence or its extract to be added to food must be studied.

It is interesting to note that in meat production, banana inflorescence has been combined with chicken flesh to create shredded banana inflorescence-chicken meat [66]. The researchers discovered that the products created with the assistance of inflorescences at a level of 25% had the highest preferences in terms of scent, taste, texture, color, and general acceptance. Banana inflorescence extract has been used in sausage and burger items. Pork patties have been treated with a 1–2 percent hydroethanolic extract of banana inflorescence [67]. The principal components, color features, pH, and sensory qualities of the burger product were not adversely affected by the addition of banana inflorescence extract. Additionally, it slowed down the oxidation of lipids in products kept at 12 °C, with the extract’s maximum concentration (2%) exhibiting the lowest levels of oxidation after 120 days of storage. Rodrigues et al. [68] used the banana inflorescence extract to create sausage, and the results showed that banana inflorescence extracts perfectly regulated lipid oxidation during storage: they can be employed as natural antioxidants in meat products. Furthermore, the product’s sensory qualities (color, flavor, and texture) were unaffected by the extract at its greatest concentration (2%).

## 4. Banana Peel

### 4.1. Fraction of Banana Peel

#### 4.1.1. Protein

The chemical features of banana peel proteins have been uncovered, including amino acid profiles (UPLC) and secondary structural properties [69]. Banana peel proteins can be further studied for their antioxidant and antifungal capabilities by isolating and purifying proteins that are unique to a target’s antifungal or antioxidant activity by acid extraction. The antioxidant activity of the acetone and ethanolic extracts of banana peel protein averaged 27.17 ± 2.69% and 27.91 ± 0.49%, respectively [69]. The protein in banana peel can be useful in commercial food or feed operations when using banana peels.

#### 4.1.2. Fatty Acids

Essential fatty acids (EFAs) have been regarded as functional ingredients and nutraceuticals. EFAs are the basis for the building blocks of cells as well as the creation of a number of physiologically active substances. Numerous research studies have demonstrated the significance of EFAs in a variety of metabolic processes [70]. EFAs have anti-thrombotic, anti-atherosclerotic, and anti-inflammatory properties. In addition, EFAs may lower the risk of major diseases like diabetes, osteoporosis, cancer, cardiovascular disease, and other diseases due to their intricate interactions with lipoprotein and cellular membrane.

According to findings from recent literature, banana peels are a good source of polyunsaturated fatty acids including linolenic and linoleic acid, which make up more than 40% of the total fatty acids. A linoleic acid-rich diet showed reduced liver fat and a slightly improved metabolic status without any signs of inflammation [71,72].

#### 4.1.3. Minerals

Studies have shown that banana peels are rich in mineral elements such as calcium, phosphorus, and potassium, which have a variety of health benefits, such as supporting organ and body function regulation, acting as coenzymes in various enzyme systems, facilitating other biochemical and physiological processes, and preventing degenerative neurological diseases [73]. Oguntoyinbo et al. [73] investigated the minerals in mature banana peel and found that calcium levels ranged from 173.33 to 178.33 mg/100 g, potassium from 75.00 to 76.67 mg/100 g, magnesium from 38.33 to 43.33 mg/100 g, and sodium from 248.33 to 250 mg/100 g. The conversion of colors in banana peels during ripening has a significant impact on the change in mineral content.

#### 4.1.4. Dietary Fiber

**Figure 5:**
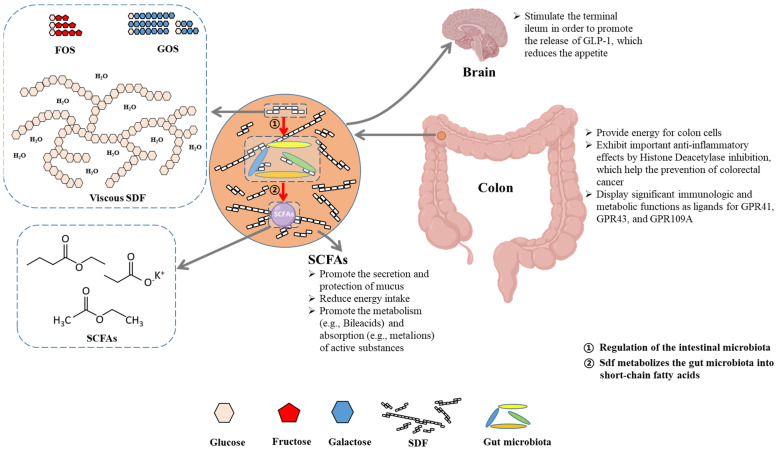
Summary of identified physiological effects of SDF and SCFAs on human health. Adapted from [74].

Dietary fiber has been shown to prevent a range of diseases including cardiovascular disease, diverticulosis, constipation, colon cancer, obesity, and diabetes [74]. The peel of a banana is a good source of dietary fiber. The two primary components of total dietary fiber (TDF) are soluble dietary fiber (SDF) and insoluble dietary fiber (IDF). IDF refers to the fiber content that is not soluble in water, such as cellulose, lignin, and certain hemicelluloses, while SDF refers to the water-soluble fraction, such as fructo-oligosaccharides (FOS), galacto-oligosaccharides (GOS), and viscous SDF [75]. In contrast to IDF, SDF can be readily metabolized by gut bacteria into beneficial products, mainly short-chain fatty acids (SCFAs), which not only reduce the risk of gastrointestinal diseases but also perform important physiological functions as ligands for GPCRs involved in immune regulation (GPR41, GPR43, and GPR109a). Figure 5 summarizes the benefits of SDF and SCFAs for human health [74]. The IDF and SDF ranged from 5.91–44.95% and 0.23–7.75% in banana peels, respectively, which indicated that IDF is the most abundant component in TDF recovered from banana peels. According to Emaga et al. [76], the TDF and IDF levels in banana peels increased from green stage to yellow.

#### 4.1.5. Polyphenols

Compared with other fruits, banana peel contains an abundance of phenolics, essential secondary metabolites [77]. Phenolic chemicals have been associated with a number of health advantages, including the reduction of obesity, diabetes, cancer, and cardiovascular disorders [77]. Polyphenols have been utilized successfully as functional additives in foods because they can stop lipid oxidation and stop the growth of bacteria and mold. In the study by Ali et al. [78], fish balls treated with different concentration total polyphenol extract from banana peel held at 4 °C and −18 °C showed a decrease in peroxide value (PV). Hydroperoxide breakdown into secondary oxidative intermediates may be the reason of the PV decline near the end of storage.

In the future, there are two clear directions for research into the use of banana peels as a cheap and abundant source of phenolic compounds: (a) the development of affordable and effective technologies for the recovery of phenolic compounds; and (b) the use of phenolic compounds as functional ingredients in food and pharmaceutical products.

### 4.2. Biofunction

#### 4.2.1. Antioxidant Activities

When compared with other fruits, banana peels have higher concentrations of phenols, an essential secondary metabolite with antioxidant characteristics. In banana peel, a number of phenolic compounds are present, including gallic acid, catechin, epicatechin, gallocatechin, and anthocyanins [79]. Additionally, the gallocatechin content of banana peel is five times higher than that of banana pulp, indicating that peel is a significant source of antioxidant compounds [80].

**Figure 6:**
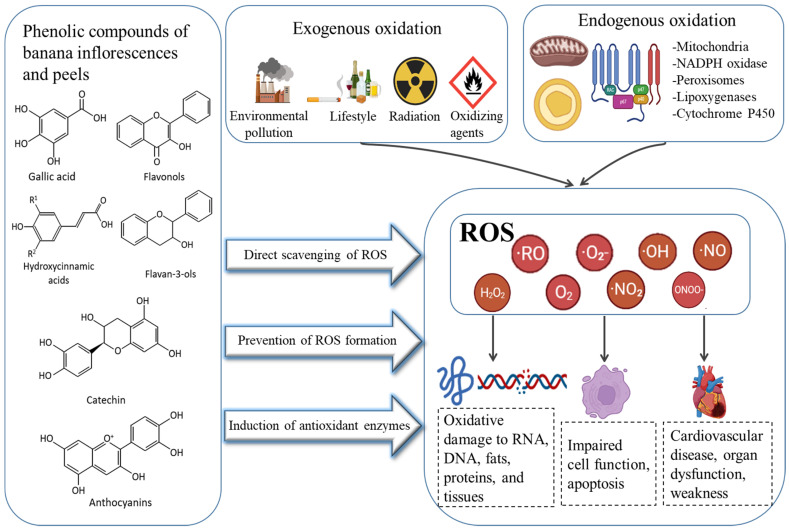
The mechanism of antioxidant action of phenolic compounds in banana by-products is proposed.

The antioxidant capacity of banana peel extracts and components is evaluated using multi-mechanistic antioxidant tests. They are crucial for determining the capacity of the chemical constituents in banana peel extracts to reduce metal ion chelation, as determined by the FRAP assay, and scavenge free radicals like ABTS and DPPH. The phenolic chemicals present in banana peels include four subgroups, which are flavonols, flavan-3-ols, hydroxycinnamic acids, and catecholamines. In the study by Vu et al. [81], the antioxidant capacity of peel increased as bananas ripened and decreased after they were overripe, demonstrating that phenolic components rather than chlorophylls and carotenoids are associated with the antioxidant qualities. Therefore, the potential applications must be assessed according to their maturity. Figure 6 illustrates how the phenolic compounds in banana by-products exert their antioxidant effects by limiting the production of reactive oxygen species (ROS), directly scavenging ROS and activating antioxidant enzymes.

#### 4.2.2. Antimicrobial Agent

Banana peel extracts have also been reported to show varying degrees of antibacterial activity. Previous investigations found that the antibacterial properties of banana peels can be used to successfully inhibit the activity of *Bacillus cereus*, *Staphylococcus aureus*, *Bacillus subtilis*, and *Escherichia coli* [82]. According to Mordi et al. [83], banana peel extract has strong antibacterial activity against a variety of microorganisms, which might be the result of 2-Methyl-5-(1-methylethyl)-phenol, a strong antibacterial molecule. The native Nigerian banana’s methanolic extracts successfully inhibited *Staphylococcus aureus*, *Pseudomonas aeruginosa*, *Escherichia coli*, *Bacillus species*, and *Klebsiella pneumoniae* [83]. Bangladeshi banana’s ethanol and ethyl acetate extracts prevented the growth of both Gram-positive bacteria (e.g., *Bacillus megaterium*, *Bacillus subtilis*, and *Staphylococcus aureus*) and Gram-negative bacteria (e.g., *Pseudomonas aeruginosa*, *Salmonella paratyphi*, and *Shigella boydi*) [84].

#### 4.2.3. Anti-Disease Activities

**Figure 7:**
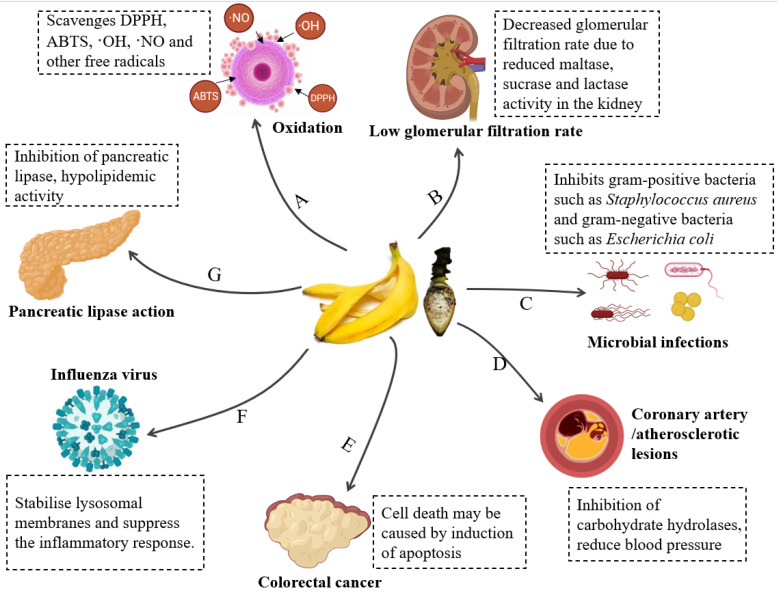
Schematic representation of the potential mechanisms of the health benefits of banana by-products and their corresponding bioactive components: (A) antioxidant, (B) antidiabetic, (C) antimicrobial, (D) anti-cardiovascular disease, (E) anticancer (F) anti-inflammatory, (G) anti-obesity.

The anti-disease benefits of banana peel actives are gaining traction. In the study by Dahham et al. [85], the extract of banana peel made from hexane solvent had the highest toxicity toward the human colon cancer cell line HCT-116, inhibiting cell multiplication by 64.02%. Durgadevi et al. [86] reported that aqueous methanol extracts of banana peels have the capacity to scavenge free radicals and against MCF-7 breast cancer cells lines. According to Vijayakumar et al. [87], banana peel extract can also be utilized to create gold nanoparticles that prevent the growth of *Enterococcus faecalis* biofilms that are cytotoxic to human lung cancer cells. The flavonoid content of banana peel is associated with its anti-cancer activity. Flavonoids inhibit the activity of ROS scavenging enzymes, induce apoptosis, and bring the cell cycle to a standstill, thereby inhibiting tumor production. The pharmacological functions and mechanisms of action in banana by-products are shown in Figure 7.

### 4.3. Comprehensive Utilization of Banana Peel

#### 4.3.1. Food Processing Raw Material

Currently, banana peels are used to enhance the physicochemical and nutritional qualities of food products such as pastries, meat products, and jellies [88]. The phytochemical and antioxidant potency of food products will undoubtedly improve with a higher banana peel concentration. However, it might result in subpar physicochemical qualities and the rejection of food goods. Therefore, it is important to research the appropriate banana peel concentrations in various food products. Table 3 lists the uses of banana peels in food manufacturing.

In order to produce fish patties, Zaini et al. [89] substituted 2%, 4%, and 6% of fish surimi with ripe banana peel, and the results demonstrated that adding banana peel pieces to fish patties can increase their cooking yield, hardness, water retention ability, and dietary fiber content. The highest overall acceptability of the goods was achieved with the replacement of banana peel at 2%. The physicochemical and sensory characteristics of Egyptian baladi flatbread were studied by Eshak [90], who used two different concentrations of banana peels (5% and 10%) in place of some of the wheat flour. The results of the chemical analysis revealed an increase in protein, fat, and ash in the Egyptian baladi flatbread. In the study by Arun et al. [91], functional cookies were created using banana flour at the ratios of 5%, 10%, and 15%, the breaking force, spread ratios, and cookie browning index all fell when the proportion of banana peel powder added increased. The DPPH scavenging activity also increased with increased banana peel powder.

**Table 3:** Benefits of banana peel and their applications.

Nutrient Content	Extraction Method	Properties of Extracted Component	Application	Purpose	References
Protein	Acid extraction (glacial acetic acid)	With antioxidant and anti-fungal properties	Food or feed	Increase the content of amino acids in food or feed	[69]
Dietary fiber	Prior to extracting the dietary fiber, tap Water was used to rinse the wet milled BP powder.	- High dietary fiber content with a 4:1 IDF/SDF ratio - High oil and water holding capacities	Chicken sausage, fish patties, cookies	To increase the amount of dietary fiber	[89,91,92,93]
Fatty acids	ND	Anti-atherosclerotic, anti-thrombotic	Drugs that prevent cardiovascular disease, cancer, osteoporosis, diabetes, and other illnesses	Increasing the content of fatty acids in food	[71,72]
Mineral	ND	- High calcium, phosphorus and potassium content - Helps maintain blood pressure and Manage heart and breathing difficulties	Used as fertilizer to supplement soil nutrients	Improve soil fertility	[94]
Pectinases	BP powder was added to a base medium that already contained yeast extract, K_2_HPO_4_, KH_2_PO_4_, and KNO_3_, following that, the samples were incubated in a solution at 37 °C while being shaken at 150 rpm.	The four lactic acid bacteria that were studied responded positively to the prebiotic effects of the banana peel powder that this enzyme hydrolyzed, suggesting that *B. amyloliquefaciens* TKU050 pectinase may be a good option for generating prebiotics	Release fibers by attacking non-cellulosic components in the sheath	get cellulose	[95]
Carbohydrate (pectin, xylose, xylooligosaccharides)	Banana peels were pre-treated with citric acid and alkaline solutions, then enzymatic hydrolysis (Cellic^®^ CTec2) was performed on the peels	The breakdown of a banana peel demonstrated that pectin, xylose, and xylooligosaccharides all necessary carbohydrates could be removed from its cell walls with a high yield	Replenishes the role of carbohydrates in the body, but also has the effect of protecting the liver and detoxification	Using Banana Peels as a Carbohydrate Supplement	[96]
Polyphenols	ND	Significantly reduces the peroxide value of fish balls	Fish ball preservative	Preservation freshness to increase the storage time of fish	[78]

Note: ND, not determined.

#### 4.3.2. Production of Feed

With the increasing human demand for food of animal origin, the demand for animal feed has risen. It is urgent to increase the productivity of feeds with higher nutritional value to overcome the problem of limited sources and high prices of raw materials for animal feed production. Banana peel is high in the main nutrients such as protein, fat, and carbohydrates, which account for 91.50% of its dry weight, and also contains a large amount of crude fiber [94]. Therefore, banana peel is one of the main raw materials for the production of animal feed.

Essien et al. [4] reported that banana peels can serve as mycological media to grow valuable microfungal biomass to enrich the protein and fatty acid content of feed. An important step in creating high nutritional quality feed from poor quality raw materials is to increase the nutritional value of banana by-products through microbial fermentation. Through microbial fermentation, the protein and sugar content of feed with added banana peel can be increased to levels comparable to soybean meal [97]. Nuriyasa et al. [98] reported that the use of 9% banana peel feed levels improved rabbit consumption, feed digestibility, growth, and live weight: the rabbits grew faster and weighed more than rabbits not fed banana peel. Banana peel used to produce animal feed might enriches the poultry feed industry.

#### 4.3.3. Fertilizer Utilization

Traditionally, banana peels were simply broken down to replenish soil nutrients and used as fertilizer. Due to the higher requirements of modern agriculture for biological fertilizers and the advancement of agricultural production, many types of organic fertilizers have been produced from banana peels [99]. Under both aerobic and anaerobic conditions, banana peel is poured into cow dung, and poultry manure and banana peel will synthesize organic fertilizer under the action of earthworms. The organic fertilizer has high potassium content (>100 g/kg) and high nitrogen content (>2%) and is effective for various plants [100].

#### 4.3.4. Energy Utilization

Banana peel is a good raw material for ethanol, biogas, hydrogen, and solid biochar production. Itelima et al. [101] used a co-culture of Brewer’s yeast and *Aspergillus niger* to ferment banana peels for 7 days; it produced 1.60 OD of biomass and the ethanol output was about 7.45% *v/v* (ranged from 0.20 to 0.82 mg/cm^3^). Banana peels can also be used to create biogas. Pisutpaisal et al. [102] analyzed gas composition and volatile fatty acid after physical treatment, and the result indicated that the methane yield of banana peel fermentation was enhanced by size reduction and fungal pretreatment. Silva et al. [103] demonstrated that the biomass in dry banana peel powders can be converted into useful syngas and conductive porous carbon using flash irradiation (biochar), produced 330 g of biochar and almost 100 L of hydrogen per kilogram of dry biomass.

#### 4.3.5. Bioremediation

Banana waste can be transformed directly or through processing into materials suitable for the elimination of metal ions. Metals such as copper, lead, cadmium, zinc, and chromium have been shown to be adsorbed by banana peel cellulose and foam carbon produced from banana peels [104]. Color removal from contaminated materials has been accomplished using banana peels and other elements [105]. Pisutpaisal et al. [102] used banana peels to remove hexavalent chromium from industrial wastewater. Chromium is a waste product from the chromium plating and metal fishing industries. Consuming large amounts of chromium can harm the kidneys, tubules, and glomeruli, and therefore, it must be removed from wastewater.

#### 4.3.6. Food Packaging

An edible food wrapper produced from banana peels by Santhoskumar et al. [106], who showed an increase in tensile strength. Due to the fiber content in banana peels that contains inorganic nutrients, the tensile characteristics were comparable with those of polyethylene, and the inorganic composition of banana peel also improved the mechanical qualities. These films are very simple to find and biodegradable. Sultan and Johari [107] found that a bioplastic film made of two polymers produced from banana peels and maize starch showed significant tensile strength. When banana peel powder was added in amounts up to 20%, it was less likely to break. The edible films created from banana peels can aid in increasing production efficiency, and they enhanced the economic benefits by creating a range of goods that utilized plastic wrapping during the manufacturing process [107].

#### 4.3.7. Pharmacological Applications

It has been discovered that banana peels contain bioactive compounds like flavonoids, phlobatannins, tannins, glycosides, alkaloids, and terpenoids that have a variety of biological and pharmacological effects, including antibacterial, antihypertensive, antidiabetic, and anti-inflammatory effects [108].

The peel of the banana has been used as a herbal medicine for a number of conditions, including burns, ulcers, coughs, and diarrhea. For instance, banana peels are used to make wound ointment to reduce burn swelling and pain [108]. Studies have found that banana peels can treat swelling and stop itching after mosquito bites [109]. The anti-acid properties of banana peel are also recognized for treating stomach ulcers. Flavonoid leucocyanidin in banana peel has been demonstrated to thicken the stomach mucosal layer [110]. The peel has also been used to treat or prevent a number of different illnesses and health issues, including anemia, blood pressure, and depression (linked to the tryptophan content in bananas). Therefore, when properly handled and processed, banana peel can offer great prospects for medicinal application.

## 5. Problems and Countermeasures in Utilization of Banana By-Products

The comprehensive use of banana by-products can not only reduce waste and environmental pollution but also enhance the economic efficiency of the banana industry. However, there are still many problems in terms of utilization efficiency and technological means and solutions. A number of difficulties have been highlighted. In transforming banana by-products into a viable agricultural commodity, the focus should always be on converting banana by-products into high-value processed raw materials or products that match market demands and have significant economic benefits [111].

### 5.1. Pesticide and Ripening Agent Residues

Banana is a fruit that is closely related to people’s lives and is subject to a variety of pests and diseases during production, which can cause serious yield losses or even crop failure [112]. Therefore, in order to ensure the production of bananas, a variety of chemical pesticides are used to control the pests and diseases. The application of pesticides in banana production will inevitably have an impact on the use of banana by-products. Among the banana by-products, banana peel is high in antioxidants and bioactivities and is often used to add nutritional value to food and to prevent some chronic diseases. It is particularly important to control and monitor the level of pesticides in banana peel if the pesticide content in the peel used for processing into food is too high for human health. Therefore, the study of the residue dynamics of pesticides in banana and developing biopesticides to reduce reliance on chemical pesticides is of far-reaching significance for the rational use of pesticides and the use of banana by-products.

### 5.2. Difficulties in Collecting

Banana by-products have been proven to have great potential for use, but due to the large volume of banana stems and leaves and the water content of more than 90%, collection and transportation from the field would be very costly. It would also require a great deal of storage space and is not easy to transport. There are currently no effective techniques and equipment for collecting, transporting, and storing banana by-products, which has led to a large amount of residues being disposed of directly in the fields [113]. The collection of banana peels is also a problem as people discard the peels directly after eating them. Therefore, the integrated use of banana by-products is a huge challenge [113].

In order to make these unprocessed materials available for industrial-scale processing, standardized collection systems and transport systems must be established. This is particularly the case with regard to the establishment of appropriate by-product collection facilities for the preservation and sorting of these by-products according to their type and quality, as well as storage systems to prevent the degradation of valuable components. It is urgent to develop a set of technologies and equipment for the collection, transport, and storage of banana waste, for the post-processing utilization of the residue resources.

### 5.3. Backwardness of Comprehensive Utilization Technology

The deep processing of banana by-products lacks processing technologies and equipment, the reliability and safety of the equipment is low, the energy consumption is extremely high, and many key technologies are yet to be broken through [114]. The collection of banana stems, leaves, and inflorescences in banana plantations requires efficient collection equipment, while the development of banana peel storage equipment is very important due to the unstable active substances within the peel. In conclusion, the development of technology and equipment for the whole process of collecting, transporting, storing, and processing banana by-products should all be given attention. The technology for the utilization of by-products is still immature, and the degree of comprehensive utilization is low, which is far from large-scale production. There is a lack of basic theoretical research on changes in by-products’ composition during storage; analysis of banana by-products’ mechanical properties, residue decomposition and soil fertility; plant nutrition; physiological metabolism; etc. [113].

### 5.4. Problems with the Properties of Banana By-Products Themselves

The properties of banana by-products themselves also limit the application of banana by-products, taking banana peel as an example. Banana peels are rich in active polyphenols, but polyphenols have poor stability and are easily oxidized. The soft texture of banana peel, rich in pectin, sugars, and tannins, not only makes it difficult to store but also makes it very susceptible to browning during processing. In addition, it may cause deterioration in nutrition and flavor, which affects the quality of the product. Therefore, the characteristics of bananas themselves make it difficult to maintain the original flavor and nutrition during processing, which affects the industrialization of the product [19]. The biofiber extracted from banana waste also degrades after a period of storage, lowering the quality of the biofiber [115]. The mechanisms of action of banana peel’s chemical components in the fight against various diseases or infections are not yet clear. The use of various methods such as genomics, transcriptomics, proteomics, and metabolomics could also provide a comprehensive understanding of the molecular processes underlying the health-promoting properties of banana peel, thus improving our understanding of banana peel phytochemicals as functional components [114]. Research is urgently needed on the physicochemical properties of banana by-products themselves in order to more fully utilize this resource.

## 6. Conclusions

Although recycling and using agricultural by-products to create commercially viable and revenue-generating products is not a new concept, maximizing the use of abundantly available resources to reduce solid waste emissions and create added value is still considered crucial. Banana by-products such as stems, leaves, inflorescences, and peels are good raw materials for the food and non-food industries, with a variety of potential applications (e.g., food additives, nutritional foods, food supplements, feed, renewable fuels, fibers, fertilizers, and pollutant absorbers). The utilization of banana by-products can have positive effects on protecting the environment and developing value-added foods for human being.

However, banana by-products safety needs to be addressed further to meet market demand. There were various techniques such as transcriptomics, genomics, metabolomics, and proteomics can be used to learn more about the molecular mechanisms of banana by-products for health and to better understand how the phytochemicals in banana by-products function as functional components. Additionally, in order to make these raw materials accessible for processing on an industrial scale, standardized processes for the collection, transportation, storage, and processing of banana by-products must be established. Creating value from waste products like banana by-products is arguably one way to ensure that ecological agriculture and food systems are sustainable and beneficial to the environmental for future generations.

## Figures and Tables

**Table 1:** Components, functions and applications of banana stems and leaves.

Nutritional Composition	Function	Applications	References
Bio-oil	Heat generation by pyrolysis, reducing greenhouse gas emissions	Biochar, soil conditioners, ethanol, biogas	[12,21]
Minerals	Essential elements for human health	Making functional bread with probiotics, organic fertilizer	[16]
Fiber	Facilitates the digestion and absorption of many nutrients and prevents many diseases	Good nutritional quality and organoleptic acceptability when added to instant noodles, feed, clothing, paper, sanitary pads	[15,18,22,23]
Polyphenols and flavonoids	Strong resistance to oxidation	Gingival depigmenting agents	[19]

**Table 2:** Benefits of banana inflorescence and their applications.

Biological Activities	Chemical Components	Extraction Method	Main Findings	Applications	References
Antioxidant activities	Total phenolics, flavonoids, tannins	70% aqueous acetone extracts	In the β-carotene linoleic acid system, extracts showed radical scavenging (ABTS, DPPH, and superoxide), and prevention of lipid peroxidation	Reduces the chance of diseases such as cardiovascular disease and diabetes. Chicken with banana inflorescence, burger	[51]
	Total phenolics	Methanol extracts	Extracts demonstrated lipid peroxidation inhibition in the β-carotene linoleic acid system, ferric-reducing antioxidant power (FRAP), and free radical scavenging activities (ABTS, DPPH)		[52]
	Dietary fiber and associated polyphenols	Water-soluble polysaccharides and fractions	Polysaccharide fractions had DPPH radical scavenging, FRAP, and metal chelating activities		[37]
	Umbelliferone and lupeol	bioassay-guided isolation of an ethanol extract	Extracts and isolated substances demonstrated reducing capability, an inhibitory impact against lipid peroxidation, and the ability to scavenge free radicals (ABTS, DPPH, and superoxide)		[53]
Anti-diabetic activities	Total phenolics; phenolic acids	extracts with water and different organic solvents	In Ehrlich ascites tumor cells, extracts had various degrees of stimulatory effects on glucose uptake	As a potential antidiabetic agent	[54]
	Gallic acid, quercetin and epicatechin	Extracts of methanol and its fractions (petroleum ether, ethyl acetate and water fractions)	Intragastric administration of the ethyl acetate part (200 mg/kg body weight/day) for 60 days resulted in a significant decrease in blood glucose levels, lipid peroxidation products		[45]
	β-sitosterol, 31-norcyclolaudenone, and (24R)-4α, 14α. 4-trimethyl-5α-cholesta-8, 25(27)-dien-3β-ol	Ethanol extract	Isolated substances prevented the production of AGEs in the BSA-fructose model and the activity of α-glucosidase and α-amylase		[55]
Anti-disease activities	GCMS profiling was used to identify steroids, fatty acids, and long chain aliphatic chemicals.	Ethanol extracts	Extracts significantly slowed the expansion of HT-29 and other cell lines	Anti-cancer drug that induces apoptosis in cancer cells	[48]
	Polyphenols	Methanol and ethyl acetate extracts	Based on information from cellular and proteomic techniques, in HT-29 cells, methanol extract resulted in apoptotic cell death		[56]
Cardiovascular protective activities	ND	Methanol and ethyl acetate extracts	LDL oxidation and ACE activity were both reduced by methanol extract.		[48]
Anti-microbial	Total phenolics	Methanol extract	Extracts showed various degrees of antibacterial activity against a number of harmful microorganisms that can be found in food	Food preservations	[57]
	polyphenols, steroids	Extracts were made using water and a variety of organic solvents, including petroleum ether, isopropanol, chloroform, ethyl acetate, hexane, methanol, and ethanol	Extracts showed variable degrees of antibacterial activity against certain Gram-positive and Gram-negative bacteria		[58]
Anti-inflammatory	Gallic acid, quercetin and epicatechin	Extracts of methanol and its fractions (water, petroleum ether, and ethyl acetate fractions)	In the liver of streptozotocin-induced diabetic rats, the ethyl acetate fraction downregulated the mRNA expression of TNF-α, TGF-β1, NF-κB, and IL-6 and suppressed the activity of 5-LOX and COX-2 in monocytes	Anti-inflammatory drugs	[45]
Anti-obesity	Total phenolics	Water extracts	Pancreatic lipase activity was reduced by water extracts	Weight loss potential	[59]

Note: ND, not determined.

## Data Availability

No new data were created or analyzed in this study. Data sharing is not applicable to this article.
